# Digital Home-Based Self-Monitoring System for People with Heart Failure: Protocol for Development of SmartHeart and Evaluation of Feasibility and Acceptability

**DOI:** 10.2196/62964

**Published:** 2025-01-28

**Authors:** Ralph Maddison, Rebecca Nourse, Reza Daryabeygikhotbehsara, Teketo Kassaw Tegegne, Paul Jansons, Jonathan Charles Rawstorn, John Atherton, Andrea Driscoll, Brian Oldenburg, Rajesh Vasa, Vassilis Kostakos, Tilman Dingler, Gavin Abbott, Paul Scuffham, Jo-Anne Elizabeth Manski-Nankervis, Dominika Kwasnicka, Finn Kensing, Sheikh Mohammed Shariful Islam, Anthony Maeder, Yuxin Zhang

**Affiliations:** 1 School of Exercise and Nutrition Sciences Institute for Physical Activity and Nutrition Deakin University Burwood Australia; 2 Faculty of Medicine Royal Brisbane and Women’s Hospital University of Queensland Brisbane Australia; 3 Faculty of Medicine University of Queensland Brisbane Australia; 4 School of Nursing and Midwifery Faculty of Health Deakin University Geelong Australia; 5 Centre for Quality and Patient Safety Research – Monash Health Partnership Monash Health Melbourne Australia; 6 Baker Department of Cardiovascular Research, Translation and Implementation La Trobe University Melbourne Australia; 7 Baker Heart and Diabetes Institute Melbourne Australia; 8 Institute for Applied Artificial Intelligence Deakin University Burwood Australia; 9 School of Computing and Information Systems University of Melbourne Melbourne Australia; 10 Faculty of Industrial Design Engineering Delft University of Technology Delft Netherlands; 11 Centre for Applied Health Economics School of Medicine & Dentistry Griffith University Gold Coast Australia; 12 Lee Kong Chian School of Medicine Nanyang Technological University Singapore Singapore; 13 Department of General Practice and Primary Care University of Melbourne Melbourne Australia; 14 Melbourne School of Population and Global Health Faculty of Medicine, Dentistry and Health Sciences University of Melbourne Melbourne Australia; 15 Department of Computer Science University of Copenhagen Copenhagen Denmark; 16 Flinders Digital Health Research Centre Flinders University Adelaide Australia

**Keywords:** smart home, health, chronic conditions, digital health, technology, behavior change, wearables, methodological considerations

## Abstract

**Background:**

Heart failure (HF) is a chronic, progressive condition where the heart cannot pump enough blood to meet the body’s needs. In addition to the daily challenges that HF poses, acute exacerbations can lead to costly hospitalizations and increased mortality. High health care costs and the burden of HF have led to the emerging application of new technologies to support people living with HF to stay well while living in the community. However, many digital solutions have not involved consumers and health care professionals in their design, leading to poor adoption. The SmartHeart project aimed to codevelop a smart health ecosystem to support the early detection of HF deterioration and encourage self-care, potentially preventing hospitalizations.

**Objective:**

This study aims to provide an overview of the SmartHeart project by describing our approach to designing the SmartHeart system, outlining its features, and describing the planned pilot study to determine the feasibility of the system.

**Methods:**

We used the Integrate, Design, Assess, and Share (IDEAS) framework to guide the development of the SmartHeart system, involving users (people with HF and their caregivers) and stakeholders (health care providers involved in the management of HF) in its design. SmartHeart is a complete remote heart health monitoring and automated feedback delivery system. It includes 2 user interfaces for patients: an Amazon Alexa conversational agent and a smartphone app. The system collects physiological, symptom, and behavioral data through wireless sensors and self-reports from users. These data are processed and analyzed to provide personalized health insights, self-care support, and alerts in case of health deterioration. The system also includes a web-based user interface for health care professionals, allowing them to access data, send messages to users, and receive notifications about potential health deterioration. A single-arm, multicenter pilot trial (N=20) is planned to determine the feasibility and acceptability of SmartHeart before evaluation through a randomized controlled trial. The primary outcome will be a description of the study's feasibility (recruitment, attrition, engagement, and changes in self-care).

**Results:**

The SmartHeart study started in January 2021 on procurement of funding. Recruitment for the pilot trial started in August 2024 and will be completed by March 2025. We have currently enrolled 12 participants. Follow-up of all participants will be completed by the end of May 2025.

**Conclusions:**

We have co-designed and developed a complete remote heart health monitoring and automated feedback delivery system for the early detection of HF deterioration and prevention of HF-related hospitalizations. The next step is a pilot study, which will provide valuable information on feasibility and preliminary effects to inform a larger evaluation trial. SmartHeart has the potential to augment existing health services and help people with HF stay well while living in the community.

**International Registered Report Identifier (IRRID):**

DERR1-10.2196/62964

## Introduction

### Burden of Heart Failure and Importance of Self-Care

Heart failure (HF) is a complex life-threatening syndrome associated with high mortality and morbidity, poor quality of life, diminished functional capacity, and substantial health care burden [1,2]. HF affects over 64 million people worldwide [1]. People with HF experience debilitating symptoms, including shortness of breath and fatigue, which significantly impact their daily activities and quality of life [3]. Beyond these persistent daily challenges, individuals experience acute exacerbations, that frequently necessitate hospitalization [4]. These hospitalizations are strongly correlated with increased mortality. In Australia, data indicates that individuals experiencing acute HF exacerbations requiring hospitalization face a 50% reduction in their remaining life expectancy (10 years for those aged 65-75 years) [5]. Research suggests that two-thirds of HF-related hospital admissions could be prevented by enhanced coordination of postacute care and improved self-care [3]. Self-care refers to the process of maintaining health through health promotion and preventive practice [3]. While international guidelines make self-care integral for HF management, people find managing their condition while living in the community difficult, and adherence to clinical guidelines is poor [3].

### Toward a Smart Health Ecosystem

There is an urgent need for new scalable models of person-centered health care in which care for long-term conditions such as HF shifts from the clinic to the home [6]. The COVID-19 pandemic was a catalyst for swift, wholesale change to the way we view and provide health care; there has been a significant increase in the use of telehealth and online care services, increased acceptance of digital health technologies by the medical community and the public, and broader recognition of the need to embrace digital health approaches to create a resilient health care system [6]. However, despite the potential for digital health to improve HF management [7], previous approaches to designing digital health interventions have largely failed to include patients and clinicians [8]. This can lead to lower levels of program adoption by users and increased dissatisfaction, stress, and nonadherence [9].

We previously argued for a smart health ecosystem, a holistic approach to support people living with long-term conditions that incorporate sensing, processing, and communication technologies [7]. Such an ecosystem uses a comprehensive network of internet-connected sensors (referred to as the Internet of Things) to collect data about the patient in the home [10,11] and processes these data to provide near–real-time individualized monitoring. It also uses predictive models to anticipate the need for medical intervention (eg, when detecting warning signs of decompensation) and support with self-care activities. While many of these discrete technologies exist, we bring them all together into a single system (named SmartHeart) to support people to better manage their HF at home and in the community. It is proposed that such a system can help maintain health outcomes, improve quality of life, promote independence, and reduce carer burden [12,13].

In this article, we present an overview of the SmartHeart project and describe its design and development approach. We provide a detailed description of the SmartHeart system and outline the planned pilot study to evaluate its feasibility and acceptability.

## Methods

### SmartHeart Project

**Figure 1:**
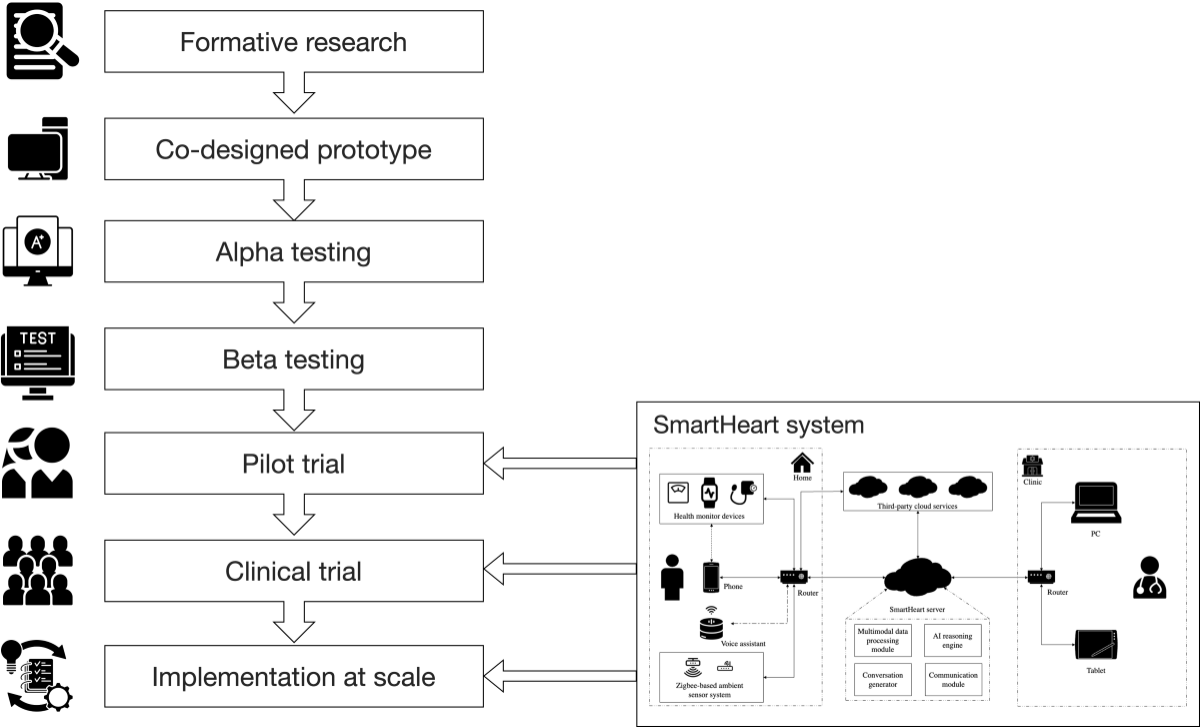
Phases of the SmartHeart project.

To guide the SmartHeart project, we used the Integrate, Design, Assess, and Share (IDEAS) framework [14]. The IDEAS framework is an intervention-specific framework for the development of digital interventions, fitting the nature of our proposed intervention [15]. Moreover, the framework combines behavioral theory with design thinking, which are complementary perspectives that can promote the acceptance and adoption of an intervention [15]. The IDEAS framework proposes ten steps: (1) empathize with target users, (2) specify target behavior, (3) ground in behavioral theory, (4) ideate implementation strategies, (5) prototype potential products, (6) gather user feedback, (7) build a minimum viable product, (8) pilot potential efficacy and usability, (9) evaluate efficacy in a randomized controlled trial (RCT), and (10) share intervention and findings [14]. In addressing steps 1-7 of the IDEAS framework, we undertook a range of studies to co-design and develop the SmartHeart system. As part of step 7, build a minimum viable product, we undertook an additional alpha- and beta-testing phase. The methods and results of these studies are briefly described in this article and the development studies are reported in full elsewhere [16,17]. The protocol for step 8 of the IDEAS framework, pilot potential efficacy and usability, is presented later in this article and will inform the design of a larger hybrid RCT to assess the effectiveness and implementation of SmartHeart, which we hope will guide subsequent delivery at scale. Figure 1 presents the phases of the overall SmartHeart project.

### Approach to Designing the SmartHeart System

**Figure 2:**
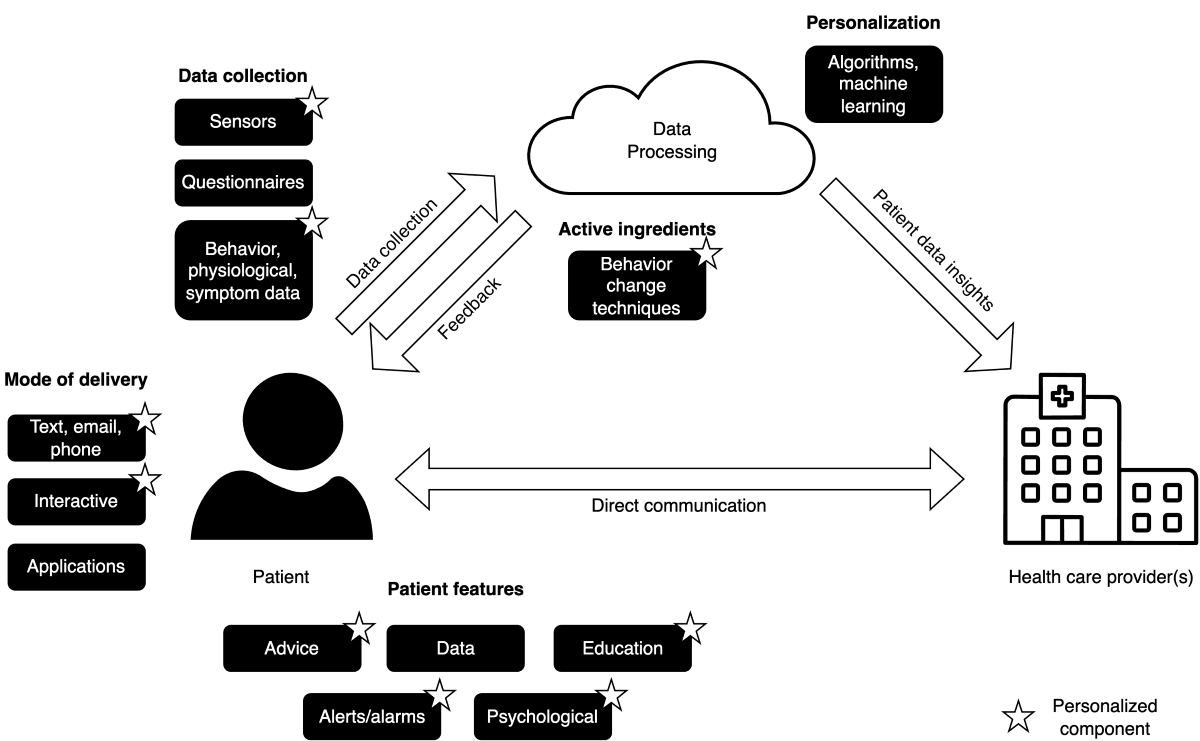
Intervention features identified in the scoping review.

As part of the formative research phase, we conducted a scoping review of existing smart health ecosystems to support people with HF [18]. This study allowed us to identify data collection, personalization, behavior change, patient-facing, and mode of delivery features of similar interventions (refer to Figure 2). These features provided us with initial ideas for the SmartHeart system.

Then, we sought to better understand the lived experiences of individuals with HF in the community and the self-care strategies they used. This involved conducting in-depth interviews with individuals living with HF (n=9) [17]. Following this, we explored potential components and features for SmartHeart from the user perspective through workshops with individuals living with HF (n=16) and their caregivers (n=4) [16]. To identify consensus-based recommendations for potential intervention features, we used the Delphi method with 15 health care providers with experience caring for people with HF (eg, general practitioners (GP), cardiologists, nurses, pharmacists, and physiotherapists) [19]. We also conducted in-depth interviews with health care providers (n=9) [20].

Building upon these findings, we refined the SmartHeart prototype through a series of co-design workshops. Specifically, we conducted 4 co-design workshops with health care providers (n=15) to gain insight into how the system would work in practice and to refine the initial prototype [16]. Subsequently, 2 further co-design workshops were conducted with people with HF (n=6) to inform the user-facing app features, and optimize the overall design look, feel, and navigation logic.

On completion of the SmartHeart prototype, we conducted alpha testing to evaluate the system’s core functionalities, user interface, and performance under controlled conditions. For alpha-testing, four members of the research and development team used the system for 6 weeks, which allowed us to assess the system's responsiveness and identify and resolve any technical issues. Then, beta testing was undertaken to assess the system's responsiveness, data accuracy, and user experience. For beta testing, 5 people with HF used the prototype system for 4 weeks. Participant feedback from this testing was used to improve system onboarding, app performance, and data visualization.

### SmartHeart System

In undertaking the design and development process outlined above, we developed the SmartHeart system (herein, SmartHeart), a smart health ecosystem to support the early detection of HF deterioration and prompt action by users with the aim of preventing HF-related hospitalizations. SmartHeart comprises components for patients (and their caregivers) and healthcare providers (ie, cardiologists, HF nurse practitioners, or other nurses).

### Patient Components

**Figure 3:**
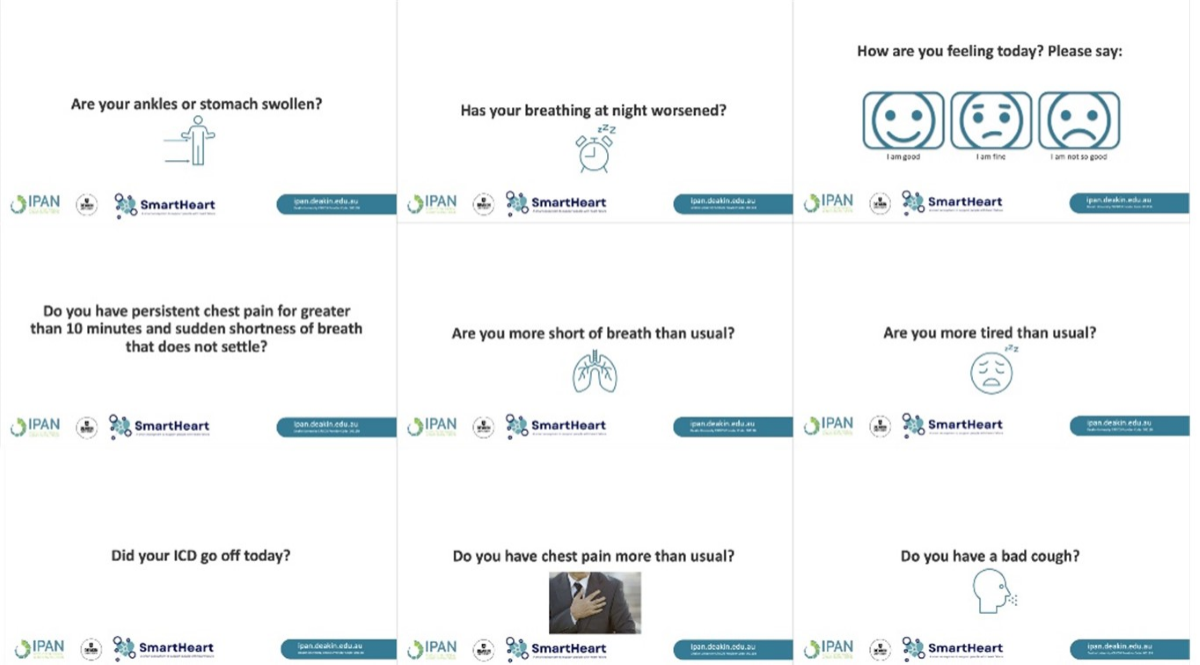
Screenshots of SmartHeart conversational agent.

**Figure 4:**
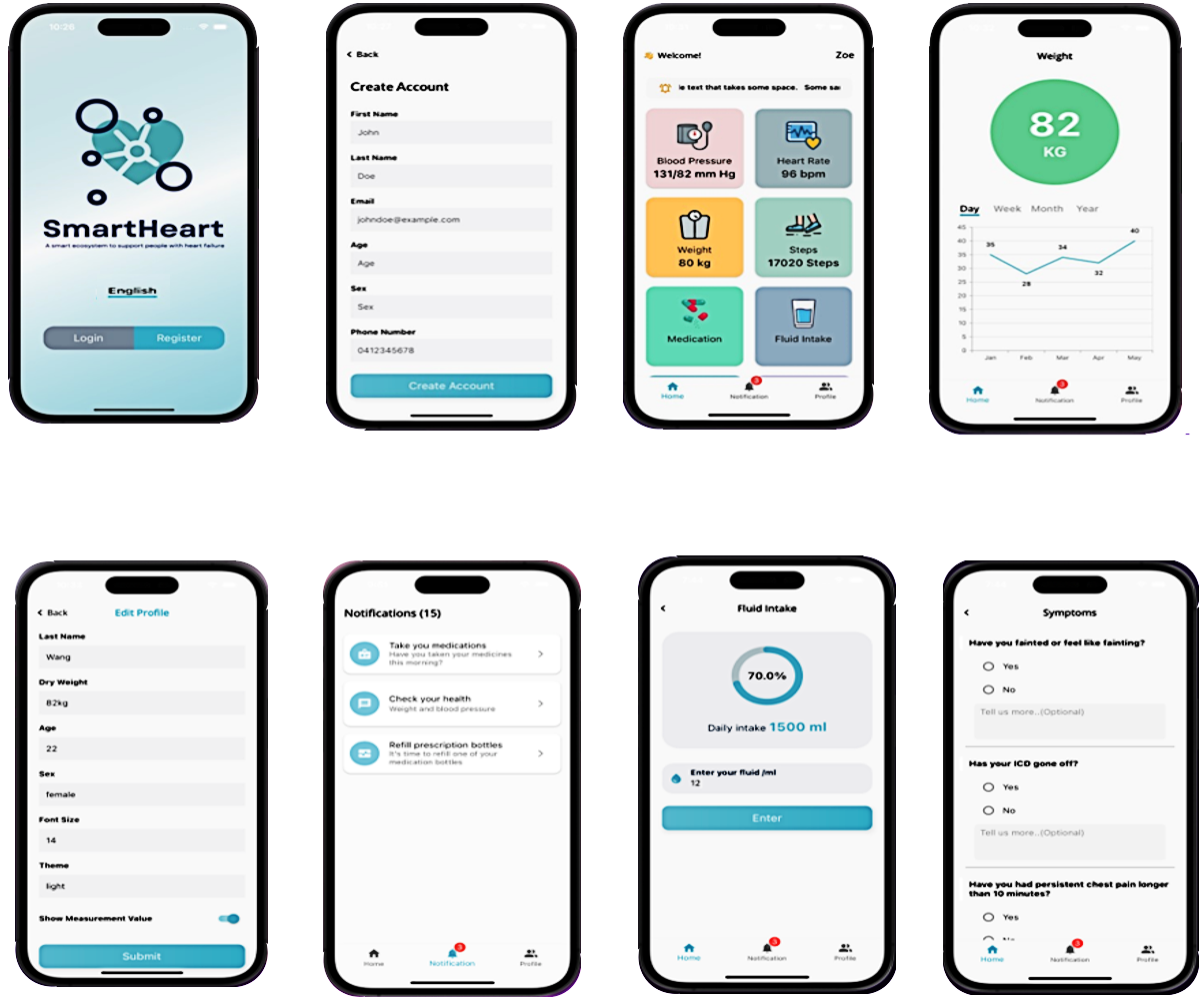
Screenshots of the SmartHeart app.

User interface: Patients and caregivers interact with SmartHeart through an Amazon Alexa Echo Show 8 (second generation), featuring an 8-inch HD touchscreen, which serves as the primary user interface and conversational agent (Figure 3). In addition, a bespoke smartphone app (Figure 4), offers an alternative interface when Amazon Alexa is not available, such as when users are away from home. Amazon Alexa was selected based on access, ease of deployment, and programmability. In its current form, Amazon Alexa can facilitate interaction with people in their own language (currently 9 languages available). The smartphone app also supports multiple languages (for initial testing, we have included Hindi and Mandarin) and time zones.

### Data Collection

SmartHeart will collect physiological, symptom, and behavioral data through wireless sensors and self-reports. The sensors included and the parameters they measure are outlined in Table 1. Users can also self-report symptoms at any time through the Amazon Alexa or smartphone app. Amazon Alexa will also periodically check in with users through timed ecological momentary assessments to gather feedback on symptoms [21]. The symptom questions are based on current practice, which involves people with HF completing a paper and pencil diary on standard symptoms (eg, shortness of breath, dizziness, cough).

To collect data on medication use, we use a proxy measure of medication access, as measured through a Philips Hue infrared motion sensor placed at the site of medication storage (eg, cupboard, drawer). This sensor will timestamp potential medication access. This approach was adopted to account for the heterogeneity of medications taken and strategies used by participants in managing their medications [16,17]. The pilot study described below will allow us to determine the feasibility of this approach. The motion sensor is connected to the Philip hub using Zigbee, a low-power wireless mesh network for connected devices. The Philips hub handles data transmission to the cloud.

**Table 1:** Wireless sensors and parameters measured.

Sensor	Parameter
Withings blood pressure monitor connects wireless blood pressure monitor	Blood pressure
Samsung Galaxy Watch Pro 5	Heart rate; activity levels (including physical activity, inactivity, and activities of daily living)
Withing’s Body Smart Bluetooth scales	Weight
Phillips Hue infrared motion sensors	Medication access (as a proxy for medication use)

### Support Features

The SmartHeart system offers a wide range of features aimed at supporting the early detection of HF deterioration and encouraging proactive self-care.

Through Amazon Alexa, users can access educational materials on topics such as healthy eating and heart health, and a home-based physical activity program. They can also set their own reminders for tasks such as taking medication, monitoring weight and blood pressure, monitoring mood, and attending health care appointments. Health data are presented to users in the form of graphs and charts, allowing them to track and self-monitor their health over time.

**Figure 5:**
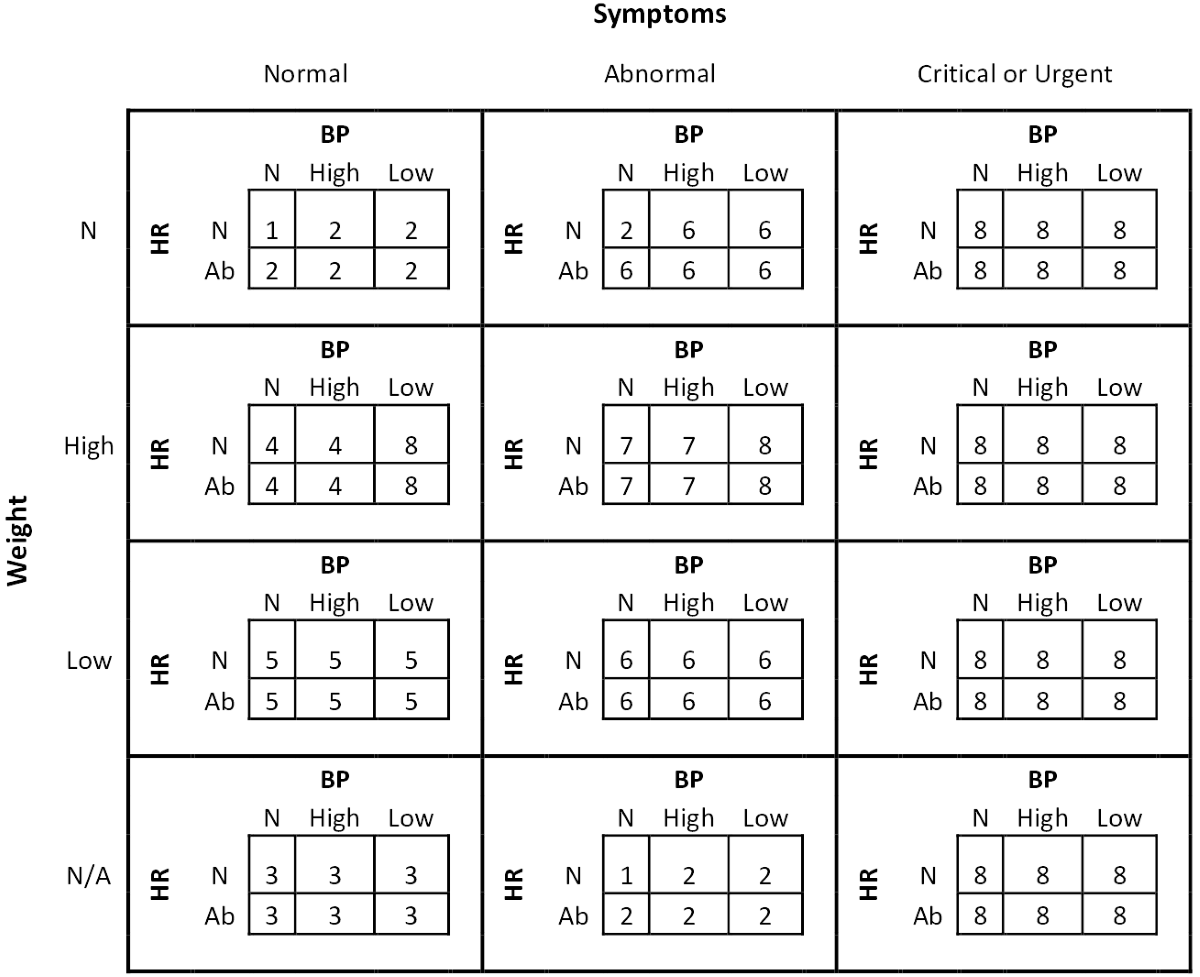
Matrix of outcome states from possible combinations of measurements. The numbers represented in Figure 5 are various patient alert messages (refer to Table 2). Ab: abnormal; BP: blood pressure; HR: heart rate; N/A: not applicable; N: normal.

SmartHeart delivers alert messages according to a matrix of possible outcome states from all combinations of sensor and symptom measurements and a corresponding rule set to define conversational agent actions. We developed the matrix based on previous work [22], but it was modified to align with the Australian HF Guidelines [2]. Alert messages and actions associated with each outcome state were validated during a face-to-face user panel workshop with health professionals (cardiologists, HF nurses, physiotherapists) at 3 hospitals (Royal Brisbane and Women’s Hospital, Bendigo Hospital, and Austin Hospital). For example, combinations of blood pressure, weight gain, and symptoms corresponding to a critical or urgent outcome state (Figure 5) will notify patients via the conversational agent (or smartphone app) to seek immediate medical support and prompt a text message to HF nurses to review data via the health care provider interface.

The numbers represented in Figure 5 are various patient alert messages (see Table 2).

Based on HF guidelines [2] and previous text messaging research in heart disease [23], we also developed a package of self-care support comprising data-driven and nondata-driven messages. In response to sensor data and self-reported symptoms, users will receive educational support and prompts to modify key self-care activities. For example, if the combinations of blood pressure, weight, heart rate, and symptoms correspond to a normal outcome state (Figure 5) users would receive personalized HF education. Alternatively, if low physical activity levels and high fluid intake were recorded, SmartHeart would prompt users to reduce fluid intake and increase their step count. This educational support and prompts will be delivered by the conversational agent or smartphone app.

In addition, users will receive regular unidirectional push notifications through the SmartHeart app only. These push notifications, delivered twice a week (1 message per day on Monday and Friday) are not data-driven and aim to encourage users to take their medication, eat a healthy diet (eg, low salt), manage their fluid intake, participate in physical activity, and reduce sedentary behavior (Textbox 1). Content for these nondata-driven notifications was adapted from our previous interventions [24,25]. Participants can opt in or out of receiving push notifications by toggling this feature on or off through the smartphone app.

**Table 2:** Examples of alert messages provided through SmartHeart.

Message number	Example message
1	“Your measurements are fine today”
2-3	“If you feel worse later today, use the system to record your symptoms”
4-5	“Contact your Heart Failure Nurse or General Practitioner (GP). Follow doctor’s orders. Restrict salt and fluids.”
6-7	“Contact your GP now or go to the emergency department if you feel you should. Follow doctor’s orders. Restrict salt and fluids”
8	“Call 000 now”

**Examples of regular SmartHeart unidirectional push notifications by category.**

**Health**

- How much time do you spend sitting every day? Decreasing the amount of time you spend sitting can improve your heart health, even if you engage in regular exercise.
- Although physical activity is useful for heart health, taking the first step can be the biggest challenge. Choose a physical activity that you like, anything that makes you more active.
- Think about barriers that can stop you from being active. Choose an activity that is more fun. Some people find it useful to invite a friend or a family member to join their daily exercise.

**Medication adherence**

- Encountering obstacles with your medication regimen? You don't need to tackle these challenges by yourself. Your doctor is there to provide assistance.
- Consistently taking your medications is essential for effectively managing your heart condition and preserving the associated benefits.
- Consistently adhering to your medication regimen is essential in managing your condition. If you believe you're having side effects, it's crucial to discuss it with your doctor. They could recommend a different medication that suits you more effectively.

**Refill prescription bottles**

- Running out of your medication could lead to a few days without it, but this is a preventable situation. Stay prepared by marking your next prescription refill date on your calendar or setting a reminder on your mobile phone.
- Ensuring the continuous availability of your medications is a crucial measure, so be certain you're aware of the upcoming refill date. Incorporating a calendar reminder can be a valuable prompt when required.
- Think about where it would be most suitable for you to store extra medications as a backup strategy.

### Health Care Provider Components

**Figure 6:**
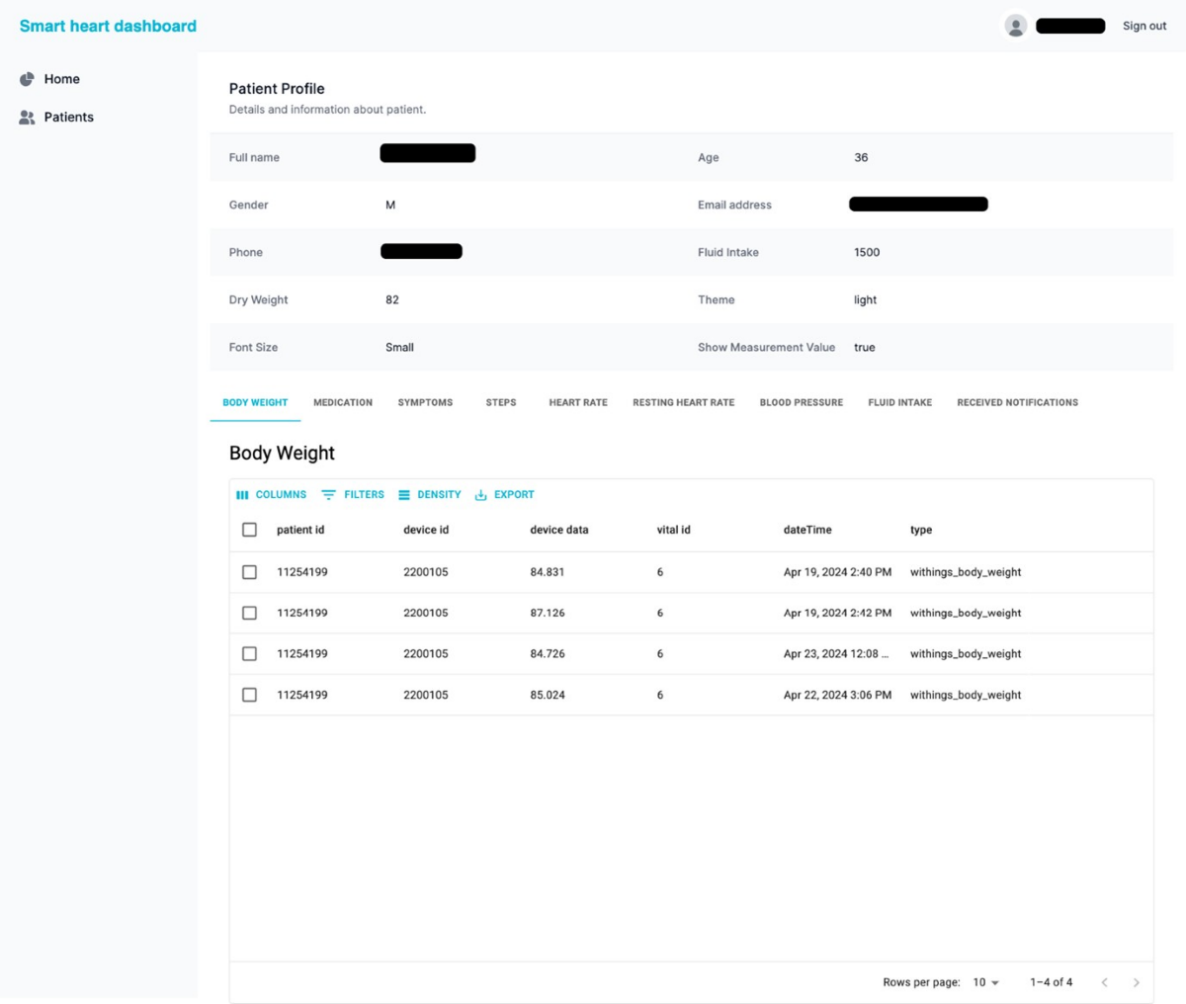
Health care provider interface.

Health care providers (ie, cardiologists, HF nurse practitioners, or other nurses) access the SmartHeart web-based user interface to register participants, review health data, and view notifications patients have received. If necessary, and according to clinical practice, health care providers will contact patients directly by telephone (Figure 6).

### System Architecture and Functions

**Figure 7:**
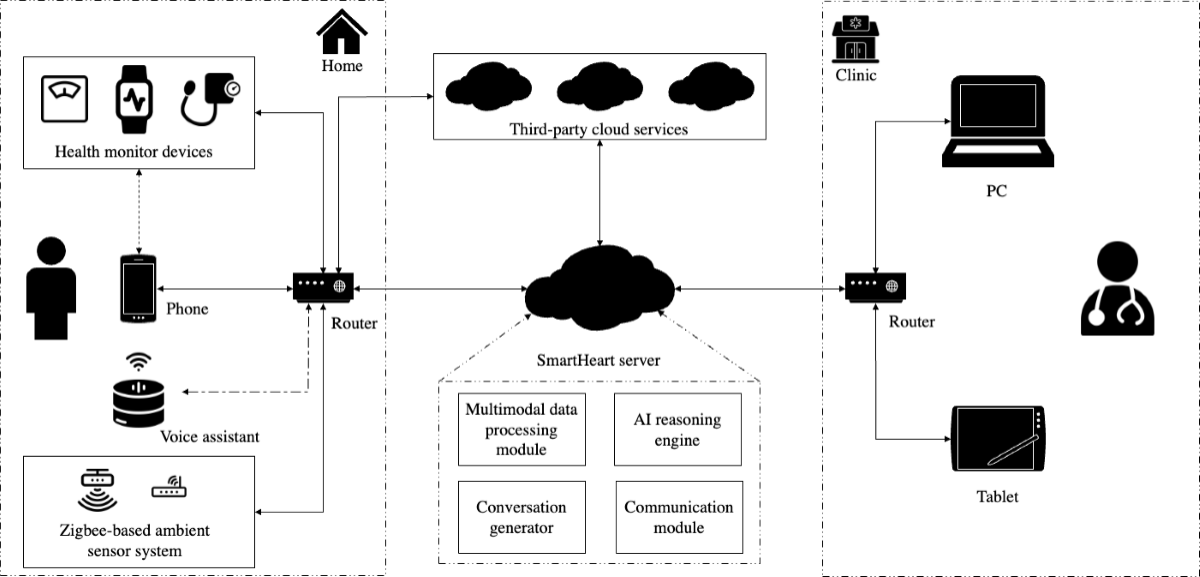
SmartHeart system architecture. AI: artificial intelligence.

Cloud-to-cloud integration connects and transmits users’ data from third-party (sensor vendor) clouds to the SmartHeart data processing server (Figure 7). This server processes multimodal data (ie, health monitoring device data and self-reported symptoms), and makes logical inferences to generate personalized recommendations for the users in different formats (ie, conversation and report). It also handles all data transmissions between end users and clinicians. Data collected through SmartHeart are stored in a secure cloud-based server hosted in Australia. All data capture, storage, and transmission are secured using state-of-the-art encryption technologies according to Health Insurance Portability and Accountability Act requirements.

### SmartHeart Pilot Study Protocol

#### Objectives

This pilot study aims to assess the feasibility and acceptability of SmartHeart and inform the design of a larger hybrid RCT to assess effectiveness and implementation outcomes.

#### Sample Size

The sample size for pilot feasibility studies should be based on practical considerations including participant flow, budgetary constraints, and the number of participants needed to reasonably evaluate feasibility goals [26]. For this study we aim to recruit a total of 20 participants, which will be sufficient to determine the feasibility of delivering SmartHeart, allowing for exploration of participant feedback regarding acceptability, usability, and uptake.

#### Participants and Eligibility Criteria

Participants will be 20 adults (aged 18 years or more) with a documented clinical diagnosis of HF, outpatients at the time of recruitment, with access to the internet, and able to read and understand the English language (for informed consent purposes) will be eligible. SmartHeart support is available in multiple languages, so people for whom English is not a first language can use it. Individuals will be excluded if they have difficulty communicating with study personnel or a conversational agent due to speech or untreated hearing problems; are planning to be away from home for ≥4 weeks during the intervention; have progressive neurological disorders including Parkinson Disease and multiple sclerosis; have schizophrenia or bipolar disorder; have renal disease requiring dialysis; have any other disorder of such severity that life expectancy is less than 12 months; or have any cognitive or physical impairment or disability that in the opinion of either the participants’ GP or specialist, or the study investigators would result in the participant having difficulty interacting with SmartHeart without support.

#### Recruitment Process

Participants will be identified by clinical staff at the 2 participating sites (Austin Health and Bendigo Health in Victoria). Research nurses at study sites will identify eligible individuals before hospital discharge and through outpatient cardiac clinics. Nurses will provide potential participants with information and consent forms and refer interested individuals to the research team. Approximately one week after the provision of study information, a researcher will confirm interest, answer questions, and schedule a baseline assessment if individuals indicate an interest in consent.

#### Procedures

SmartHeart will be installed in participants’ homes and demonstrated by a trained researcher. Written, verbal, and audio-visual support will be provided to help participants engage with the system. A loan phone (Android) will be provided free of charge to those who do not own one. With the help of SmartHeart, participants will be asked to record daily measurements of weight and blood pressure and answer questions about their symptoms. All participants will have access to their usual cardiology and general practice care throughout the course of the study.

For this study, HF nurses will have access to the clinician portal through which they can review participants’ data, including notifications. HF nurses will receive email notifications to alert them of potential changes to the participants’ HF condition. Australian HF guidelines and best practices will be followed when responding to notifications. HF nurses will be available to review sensed data and respond to notifications during their working hours; however, SmartHeart will alert participants to contact their GP as first contact.

### Data Collection

Data will be collected at baseline and the end of the 8-week intervention period using self-reported questionnaires and qualitative interviews (refer to Table 3 for an overview).

In addition, to the items in Table 3, a qualitative researcher will conduct one-to-one in-depth interviews with all participants, their caregivers (if applicable), and health care providers involved in the delivery of SmartHeart. Interviews will be based on the UK National Institute for Health and Care Excellence (NICE) Evidence framework for digital health technologies [27] and will address acceptability, demand, practicality, frequency of monitoring, burden to participants, potential of provoking anxiety, scenarios in which the program would be best employed, compliance with functional components, facilitators, and barriers to participation.

**Table 3:** Summary of outcome measures.

Items	Description or tool used to measure the item	Baseline	During intervention	Postintervention (8 weeks)
Recruitment and attrition	Study recruitment will be recorded by a trained research assistant and will include the total number of participants referred to the study, numbers who agree to participants, and those who enroll in SmartHeart. The research assistant will also record the number of participants that complete the study as a measure of attrition.	✓	✓	✓
Engagement	System use logs will be collected through the SmartHeart system and will provide data on the frequency of use of SmartHeart features. These will not require user input.	—^a^	✓	—
Weight	Withings Body Smart Bluetooth scales	✓	✓	—
Physical activity	Samsung Galaxy Watch Pro 5	—	✓	—
Heart rate	Samsung Galaxy Watch Pro 5	—	✓	—
Blood pressure	Withings blood pressure monitor Connect wireless blood pressure monitor	—	✓	—
Symptoms	Self-reported HF symptoms through the SmartHeart app	—	✓	—
Self-care	Self-Care of Heart Failure Index [28], version 6 [29]	✓	—	✓
Usability	System Usability Scale [30]	—	—	✓
Health-related quality of life	European Quality of Life 5D 5-level questionnaire [31]; Minnesota Living With Heart Failure Questionnaire [32]	✓	—	✓
Medication adherence	Philips Hue Infrared Motion Sensor (proxy); Medication Adherence Rating Scale [33]	✓	✓	✓
Hospitalization	Data provided by sites or study nurses.	—	✓	✓

^a^Not applicable.

### Self-Report Measures

Self-Care will be assessed using the Self-Care Heart Failure Index (SCHFI) [28], version 6 [29]. The SCHFI comprises 22 items and collects data on 3 domains (self-care maintenance, management, and confidence). Scores can be summed, but this is discouraged by the authors of the SCHFI. They recommend that the scales (maintenance, management, confidence) be used individually. Each scale is standardized to a score of 100. A score of ≥70 can be used as the cut-point to judge self-care adequacy, although evidence is provided that benefit occurs at even lower levels of self-care [29].** **The reliability and validity of this measure have been well documented [29].

The System Usability Scale (SUS) comprises 10 items (each question with a Likert scale ranging from strongly agree to strongly disagree) and was designed as a “quick and dirty” measure of the usability of either hardware or software or both by end users’ system [30]. Five questions are positively framed, and 5 questions are negatively framed. The process for computing an SUS score is as follows: (1) subtract 1 from the user’s Likert ratings for odd-numbered items or questions, (2) subtract the user’s Likert ratings from 5 for even-numbered items, (3) each item score will range from 0 to 4, and (4) sum the numbers and multiply the total by 2.5. This calculation will provide a range of possible SUS scores from 0 to 100.

Previous research has shown that a mean score of 68 is a useful benchmark [34].

Health-related quality of life will be measured using the European Quality of Life 5D 5-level (EQ-5D-5L) questionnaire [31] and the HF-specific Minnesota Living With Heart Failure Questionnaire (MLWHFQ) [32].

The EQ-5D-5L comprises 5Ds: mobility, self-care, usual activities, pain or discomfort, and anxiety or depression. Each dimension has 5 levels: no problems, slight problems, moderate problems, severe problems, and extreme problems. Participants are asked to indicate their health state by ticking the box next to the most appropriate statement in each of the 5Ds. This decision results in a 1-digit number that expresses the level selected for that dimension. The digits for the 5Ds can be combined into a 5-digit number that describes the patient’s health state. It also includes a visual analog scale that captures the participant’s self-rated health. This can be used as a quantitative measure of health outcomes that reflects the participant’s own judgment. The EQ-5D-5L has demonstrated excellent psychometric properties across a broad range of populations and health conditions [35].

The MLWHFQ is a 21-item multidimensional questionnaire designed to assess the health-related quality of life of people with HF in adults across physical, socioeconomic, and emotional or psychological dimensions. It is scored through a 6-point Likert scale (0-5), with a sum of item responses for total and dimension scores. The MLWHFQ has very good psychometric properties [36].

The Medication Adherence Rating Scale [33] is a 10‐item self‐report adherence scale that assesses both intentional (“I avoid using it if I can”) and nonintentional medication nonadherence (“I forget to use it”). Psychometric properties are well established [37]. Scores range from 0-10 with higher scores indicating better attitudes toward medication.

### Feasibility Outcomes

To inform decisions to progress to a definitive trial, there must be evidence of achieving key criteria for recruitment, engagement, self-care, and attrition. For this, the criteria in Textbox 2 will be used. These criteria were not based on published data but were developed following expert discussion with the chief investigator team, which comprised 2 cardiologists, an HF nurse specialist, 2 public health researchers, 2 exercise physiologists, one computer scientist, a health economist, and a biostatistician.

**Feasibility criteria and metrics.**

Green: If all four green criteria are met, we will consider the study feasible (unless there is a clear indication from the qualitative interviews and our experience that would improve the study)

- At least 80% of the target sample size is recruited within 3 months.
- At least 50% of participants engaged with SmartHeart.
- At least 60% of participants maintained or improved their Self-Care of Heart Failure Index score.
- Attrition ≤20%. A rule of thumb states that >20% attrition poses threats to study validity [38].

Amber: If one or more of our amber criteria are met, we will consider the study as likely feasible but will consider the results of the feedback from the qualitative interviews and our experience to improve whichever criteria are not at the “green light” level before considering a full trial.

- 50%-79% of the target sample size is recruited within 3 months.
- 35%-49% of participants engaged with SmartHeart.
- 45%-59% of participants maintained or improved their Self-Care of Heart Failure Index score.
- Attrition 20.1%-35%.

Red: If one or more red criteria are met, we would consider the study as likely not feasible with the current protocol.

- <50% of the target sample size is recruited within 3 months.
- <35% of participants engaged with SmartHeart.
- <45% of participants maintained or improved their Self-Care of Heart Failure Index score.
- Attrition >35%.

### Analysis

Descriptive statistics (mean and SD for continuous data, N and percentage for categorical) will be presented for all quantitative primary and secondary (eg, self-care behaviors, Health-Related Quality of Life) outcomes and will be the primary form of analysis. The EQ-5D-5L questionnaire will be scored using the Australian value set [39]. Within a person, cross-correlation matrices will also be generated [40].

Interviews will be audio-recorded and transcribed. We will use framework analysis to analyze the interviews, using predefined categories according to the NICE Evidence framework for digital health technologies [27].

### Ethical Considerations

The study received ethical approval from the Deakin University Human Research Ethics Committee (HREC/76317/MH-202). A participant information sheet and consent form will be sent to participants during the baseline assessment. Written informed consent will be obtained at the baseline assessment before data collection.

## Results

The SmartHeart project, including the proposed pilot study, received funding from the National Health and Medical and Research Council grant (grant number 2018698) and commenced in January 2021. This study was conducted in collaboration with health and academic partners in the states of Victoria and Queensland, Australia. Recruitment for the pilot trial started in August 2024 and will be completed by March 2025. We have currently enrolled 12 participants. Follow-up of all participants will be completed by the end of May 2025. Once analysis from the pilot study has been completed, we will submit the findings for publication.

## Discussion

### Principal Findings

This article reports on the formative development of the SmartHeart system, a smart health ecosystem with advanced telemonitoring and behavioral support, offering a comprehensive, integrated, and coordinated approach to HF management. We also outline a protocol for piloting SmartHeart to inform a future RCT.

Consistent with the need to integrate consumers in health research [41], we have undertaken extensive user-centered design and engagement with end users. A comprehensive program of research, following the IDEAS framework was used in developing SmartHeart. Reflecting on the design and development process, we benefited greatly from using the IDEAS framework. Our formative work involving a scoping review, stakeholder interviews, and a Delphi survey was important for ideation and informing the prototype features and functions. Co-design workshops were critical in identifying the needs of users and health care providers. We hypothesize that this will likely maximize the acceptability, engagement, and usability of the SmartHeart system. However, the downside to this co-design approach was the much longer development timeframes to achieve the SmartHeart prototype.

SmartHeart incorporates a conversational agent (Amazon Alexa). With ongoing development, conversational agents have evolved into digital systems that aid the delivery of health interventions for individuals at the places most convenient for them. Using voice to interact with these agents is a more natural user interface than traditional mouse, keyboard, or touch interfaces, which may lower barriers to entry for many people, especially older adults, those culturally and linguistically diverse, and people with low literacy [42]. We also provide access to SmartHeart in multiple languages, increasing the accessibility of support to those who speak languages other than English. Furthermore, in Australia, SmartHeart could have a significant impact by providing greater access to health care, especially in high-priority rural and regional areas.

There are some limitations of the current system that warrant consideration. First, the system is currently Android-based; however, we offer participants an Android phone for the duration of the study, and we are developing an iOS version for future use. Second, the Samsung Galaxy watch used to collect data from participants is also only compatible with the Android system. Our future plans are to support most off-the-shelf smartwatches in the market, including Fitbit (Google; iOS and Android), Apple Watch (iOS), and Garmin. Third and finally, we use a cloud-to-cloud approach, which can be problematic when third-party providers (eg, Withings) provide updates affecting data capture. Our team will check and maintain the system on a regular basis to ensure all components are up to date, helping to mitigate this issue.

A risk mitigation strategy plan was developed for the funding application that supported this project. A key risk is the slow recruitment of participants. Our current recruitment strategy includes recruitment from participating partner hospital sites; however, we will mitigate risk by also recruiting from community HF support groups, general practices, and online through social media. The small number of participants may also be a risk as this may not be sufficient to inform feasibility; however, we have indicated in our ethics application that we may extend recruitment to mitigate this risk. Data security is another risk, which will be addressed by ensuring all data are anonymized, and data storage meets Melbourne and Deakin’s Research Conduct Policy and the Research Data and Primary Materials Management Procedure. As mentioned earlier, all data will be stored securely on password-protected servers and in accordance with ethical procedures. Finally, because participants are required to provide consent in English, it is possible that the benefit of SmartHeart being available in different languages will not be realized. The requirement for consent in English is based on logistics; we do not have a resource to pay for interpreters; however, most participants in Australia for whom English is not a first language can read English for consent purposes.

The SmartHeart system has significant potential for future expansion and enhancement. While our current prototype uses desktop smart speakers, the integration of conversational agents on mobile devices (eg, phones, tablets, smartwatches) could extend the system’s reach beyond the home environment. Furthermore, we are exploring the integration of generative artificial intelligence to enhance user engagement through more natural dialogue and personalized responses, leveraging a curated guideline-based database and retrieved augmented generation techniques. While the project’s initial focus targets people living with HF, the system’s architecture supports broad applicability to other long-term conditions. This versatility will be particularly beneficial for the increasing number of people managing more than one long-term condition. The system's adaptability is enhanced by the fact that challenges associated with HF significantly overlap with other health conditions, such as other cardiovascular conditions, chronic obstructive pulmonary disease, and diabetes, making the core functionality transferable. Potential adaptations include customizing symptom questionnaires for one or more conditions, implementing different wireless sensors, and refining the algorithms and support messages.

The next step is to assess the feasibility of this version of the SmartHeart system with both people with HF and health care providers. A pilot study will provide valuable information on feasibility and preliminary effects, which will inform the design of a larger proposed RCT. If clinical and cost-effectiveness are subsequently demonstrated, SmartHeart could augment existing health care services to support people with HF to stay well living in the community and living independently. The primary benefit of this solution is to empower people with HF to be more actively engaged in self-care and realize the unfulfilled potential for digital health to transform health care delivery. If proven feasible, it could improve clinical outcomes (hospital admissions, self-care, health-related quality of life) and reduce the cost burden of the health care system. The SmartHeart concept aligns with recent calls for a health care shift from clinics to the home, with digital health support [6] and a recent European Society of Cardiology position statement [43] to address key elements of digital health implementation.

**Conclusion**

The SmartHeart system represents a promising, user-centered approach to HF management through advanced telemonitoring and behavioral support, with significant potential for expansion to other chronic conditions. Future research, including a pilot study and larger RCT, will be crucial in assessing its feasibility, effectiveness, and impact on both clinical outcomes and health care costs.

## Abbreviations

EQ-5D-5L questionnaire: European Quality of Life 5-Dimension 5-Level questionnaire
GP: general practitioner
HF: heart failure
IDEAS: Integrate, Design, Assess, and Share
MLWHFQ: Minnesota Living With Heart Failure Questionnaire
NICE: UK National Institute for Health and Care Excellence
RCT: randomized controlled trial
SCHFI: Self-Care of Heart Failure Index
SUS: System Usability Scale

## Data Availability

The datasets generated and analyzed in this study will be made available upon publication of the principal findings.
